# Enhancement of Radiation Sensitivity in Lung Cancer Cells by a Novel Small Molecule Inhibitor That Targets the β-Catenin/Tcf4 Interaction

**DOI:** 10.1371/journal.pone.0152407

**Published:** 2016-03-25

**Authors:** Qinghao Zhang, Mei Gao, Guifen Luo, Xiaofeng Han, Wenjing Bao, Yanyan Cheng, Wang Tian, Maocai Yan, Guanlin Yang, Jing An

**Affiliations:** 1 Department of Pharmacology, State University of New York, Upstate Medical University, Syracuse, New York, United States of America; 2 SUNY Upstate Cancer Research Institute, State University of New York, Upstate Medical University, Syracuse, New York, United States of America; 3 Department of Medicine, Liaoning University of Chinese Traditional Medicine, No. 33 Beiling Street, Huanggu District, Shenyang, China; 4 Sanford Burnham Prebys Medical Discovery Institute, La Jolla, California, United States of America; 5 Department of Medicine, University of California San Diego, La Jolla, California, United States of America; University of Kentucky, UNITED STATES

## Abstract

Radiation therapy is an important treatment choice for unresectable advanced human lung cancers, and a critical adjuvant treatment for surgery. However, radiation as a lung cancer treatment remains far from satisfactory due to problems associated with radiation resistance in cancer cells and severe cytotoxicity to non-cancer cells, which arise at doses typically administered to patients. We have recently identified a promising novel inhibitor of β-catenin/Tcf4 interaction, named BC-23 (C_21_H_14_ClN_3_O_4_S), which acts as a potent cell death enhancer when used in combination with radiation. Sequential exposure of human p53-null non-small cell lung cancer (NSCLC) H1299 cells to low doses of x-ray radiation, followed 1 hour later by administration of minimally cytotoxic concentrations of BC-23, resulted in a highly synergistic induction of clonogenic cell death (combination index <1.0). Co-treatment with BC-23 at low concentrations effectively inhibits Wnt/β-catenin signaling and down-regulates c-Myc and cyclin D1 expression. S phase arrest and ROS generation are also involved in the enhancement of radiation effectiveness mediated by BC-23. BC-23 therefore represents a promising new class of radiation enhancer.

## Introduction

Despite recent advances in the delivery of radiotherapy and chemotherapy for locally advanced lung cancer, most patients relapse and succumb to their disease [1–3]. This may be due, in large part, to the presence of lung cancer stem cells: a population of cells that is capable of self-renewal, proliferation, and metastasis and that shows appreciable radioresistance [4–6]. Cisplatin and paclitaxol are the two drugs most widely used in patients to sensitize lung cancer cell to radiation therapy [7]. However, the side effects and resistance to these drugs still present barriers for improving their therapeutic indexes.

Non-small cell lung cancers (NSCLCs) account for 85% of human lung cancer cases [8]. Investigations are ongoing on several new classes of small molecule radiosensitizers and their radiation enhancing effects on NSCLCs and other human cancers [9–12]. At present, a critical need remains for the discovery and development of novel radiation enhancers that show high efficiency and low toxicity.

Aberrant activations of the Wnt/β-catenin signaling, which result in up-regulation of self-renewal and proliferation of lung cancer cells, are critical for lung cancer tumorigenesis, progression, and chemo- and radioresistance [13–15]. The Wnt/beta-catenin pathway is activated in 75% of all clinical NSCLC cases tested and plays a critical role in cell proliferation and survival [16, 17]. This pathway is over-activated in NSCLC and many other cancers due to overexpression of Tcf4, Wnt1, and Wnt2 and leads to an elevated accumulation of β-catenin in nuclei [18–20]. β-catenin binds to members of the Tcf/Lef family, regulating the expression of target genes such as c-Myc and cyclin D1 [21–23]. Inhibition of the overexpression of Wnt 1, Wnt 2, and β-catenin leads to *in vitro* NSCLC cell apoptosis and diminished *in vivo* tumor mass [20].

Emerging evidence implicates the Wnt/β-catenin pathway in the radioresistance of cancer cells [22, 24]. Nuclear β-catenin and Tcf4 accumulations or Wnt/β-catenin pathway hyper-activation are important causes of radioresistance [25]. Silencing of Tcf4 causes a significant sensitization of cancer cells to low doses of radiation [26]. An inhibitor of Wnt/β-catenin signaling pathway, GDK-100017, has been reported to enhance radiosensitivity of NSCLC cells by blocking the β-catenin-Tcf/Lef interaction [24].

Cancer stem or initiating cells that have elevated levels of nuclear β-catenin can evade the cell death normally induced by radiation. This is partially ascribed to the action of β-catenin, together with its downstream genes, c-Myc and cyclin D1, which mediate the upregulation of self-renewal and maintenance of cancer stem/progenitor cells against sublethal or lethal stimuli [22, 27]. Inhibition of Wnt/β-catenin signaling reduces c-Myc and cyclin D1 levels, thereby enhancing the radiosensitivity of cancer cells [24, 28, 29], but the precise regulatory relationships among β-catenin, c-Myc, cyclin D1, reactive oxygen species (ROS), and cell cycle arrest/progression require further clarification. Nevertheless, the specific disruption of the interaction between nuclear β-catenin and Tcf4 following selective radiation treatment represents a particularly promising strategy for preventing the proliferation and survival of cancer cells. This strategy also preserves the beneficial function of β-catenin interactions with other physiological ligands [30].

In the present study, we report on a new and potent radiation enhancer, BC-23 (C_21_H_14_ClN_3_O_4_S), which targets β-catenin/Tcf4 interaction and signaling. At 3 μM, which is a dose that causes little cytotoxicity, BC-23 treatment causes strong synergistic enhancement of the cancer cell death induced by low doses of radiation (i.e., a 2 log enhancement of cancer cell death after combination with radiation). Down-regulation of c-Myc expression, up-regulation of ROS production, and abrogation of G2/M arrest are the molecular mechanisms underlying the radiation-enhancing effects of BC-23. This report is the first to describe the overcoming of radioresistance in human NSCLC cells using a small molecule inhibitor of β-catenin/Tcf4. These data suggested a potential usefulness and application of this compound for the treatment of lung cancer.

## Materials and Methods

### Reagents and cell culture

BC-23 (NSC45382) was obtained from the NCI database. ICRT14, N-acetyl cysteine (NAC), and H_2_DCFDA, were purchased from Santa Cruz Biotechnology, Sigma-Aldrich, and Cayman-Chemical, respectively. H1299 and H1975 cells were purchased from ATCC. The cells were cultured and maintained in a humidified 5% CO_2_ atmosphere at 37°C in RPMI1640 (Hyclone, Thermal Scientific) supplemented with 10% fetal bovine serum, 2 mM glutamine, 100 U/mL penicillin and 100 μg/mL streptomycin.

### Fluorescence polarization-based competitive binding assay

Fluorescence polarization (FP) was performed according to our previous reports, with minor modifications [31]. Briefly, 20 nM tracer [FITC labeled Tcf4_(8–30)_ probe] and 500 nM β-catenin were mixed and incubated with/without BC-23 in 100 μL PBS (containing 0.01% Triton X-100). After 3 h incubation at room temperature, FP values were recorded using a Synergy 2 microplate reader (BioTek) at an excitation/emission of 485/528nm.

### TOP-flash Luciferase reporter gene assay

H1299 cells at 1×10^4^ cells/well were transfected with the nt/β-catenin reporter gene TOP-flash, harbors Tcf4 binding sites for β-catenin and the pRL-CMV Renilla-luciferase control reporter vector (Promega, E2261). Four hours after transfection, cells were treated with various concentrations of BC-23 or ICRT14. The luciferase activity was determined 24 h after the treatment using the Dual-Luciferase® Reporter Assay System (Promega, E1910). TOP luciferase activity was normalized by control pRL-CMV Renilla Luciferase activity.

### *In silico* virtual screening

We prepared the receptor structure from a crystal structure of β-catenin/TCF4 complex (PDB code 1JPW) and performed an *in silico* virtual screening using Autodock4 as previously reported [30].

### Real-Time PCR

H1299 cells were treated with different concentrations of BC-23 for 16 h. Total RNA was isolated using an RNeasy Kit (Qiagen). The cDNA was synthesized using a high-capacity cDNA reverse transcription Kit (Invitrogen). Real-time PCR was performed on a LightCycler^®^ 480 System using LightCycler^®^ 480 SYBR Green I Master (Roche). The threshold cycle (C_t_) values were normalized to β-actin internal reference. The primers used in real time PCR were as follows: β-actin, forward 5′-AGAAAATCTGGCACCACACC-3′; reverse 5′-AGAGGCGTACAGGGATAGCA-3′; c-myc, forward 5′-TCAAGAGGCGAACACACAAC-3′; reverse 5′-GGCCTTTTCATTGTTTTCCA-3′; cyclin D1, forward 5′-AACTACCTGGACCGCTTCCT-3′; reverse 5′-GGGGATGGTCTCCTTCATCT-3′.

### Cytotoxicity assay

H1299 cells, plated in 96-well plates at 1x10^3^ cells/well were treated with the desired concentrations of BC-23 in 100 μL medium, and incubated for 1 day or 3 days. Cell viability was determined by adding 20 μL CellTiter-Blue (Promega, G8081) to each well. After a 4 h incubation at 37°C, fluorescence was determined using a Synergy 2 microplate reader (BioTek) at an excitation/emission of 550 nm/600 nm.

### Clonogenic assay

H1299 cells were irradiated with 0–10 Gy radiation (RS-2000 Biological Research Irradiator, Rad Source). One hour later, cells were treated with DMSO or 3 μM BC-23 and incubated for another 24 h. The cells were then seeded in 6-well plates at 500–2000 cells per well, and incubated for 12 days to allow colony formation. Colonies consisting of 50 or more cells were counted under a microscope. The plating efficiency (PE) was calculated by dividing the number of colonies by the cell number seeded. The surviving fraction was calculated by dividing the PE values of radiation groups by that of the corresponding non-irradiated groups. The effect of the antioxidant NAC on BC-23-induced cytotoxicity and inhibition of growth in H1299 cells was determined after adding 1 mM NAC, along with various concentrations of BC-23, to the cells. After incubation for 24 h, cytotoxicity and colony formation were determined as described above.

### Transient transfection with CTNNB1 siRNA

H1299 cells were plated in six-well plates. Upon reaching 50% to 60% confluence, the cells were transfected with validated human b-catenin (CTNNB1) siRNA or negative control siRNA 1 (Ambion, Inc.) at a final concentration of 100 nM with JetPRIME reagent (Polyplus transfection, NY USA), as per the manufacturer’s instructions. After a 24 h transfection, cells were split into 2 groups, one for western blots and the other for radiation sensitivity analysis. For western blots, the culture medium was replaced with newly prepared medium and the cells were incubated for another 24 h before protein preparation. For radiation sensitivity analysis, the cells were treated with or without 6 Gy radiation. After radiation, the cells were treated with 3μM BC-23 or the DMSO vehicle and harvested at 48 h after transfection for clonogenic assays.

### Protein extraction and western blot analysis

The cells were collected and lyzed in lysis buffer (50 mM Tris, pH 7.5, 150 mM NaCl, 1 mM EDTA, 0.1% sodium dodecyl sulfate (SDS), 1% deoxycholic acid, 2 mM orthovanadate, 100 mM NaF, 1% Triton X-100, 0.5 mM phenylmethylsulfonyl fluoride) containing a protease inhibitor and phosphatase inhibitor cocktail (Sigma-aldrich). Protein contents were quantified by the BCA method. Protein samples (20μg) were electrophoresed in 10% SDS polyacrylamide gels and transferred to polyvinylidene difluoride membranes (Millipore, Bedford, MA, USA). The membranes were blocked and incubated overnight at 4℃ with a monoclonal rabbit anti-β-catenin (1:1000, Cell Signaling Technology, MA, USA) primary antibody, or with a monoclonal mouse anti-β-actin (1:2000, Sigma–Aldrich, St. Louis, USA) primary antibody as a loading control. After washing, the blots were incubated for 1 h at room temperature with corresponding horseradish peroxidase (HRP)-conjugated secondary antibodies (1:5000; Santa Cruz Biotechnology, CA, USA), visualized with ECL solution (Super Signal West Pico Chemiluminescent Substrate, Pierce, Rockford, IL, USA), and exposed using the ChemiDoc™ MP System (Bio-Rad).

### Cell cycle analysis

H1299 cells were plated in 6-well plates and irradiated with 4 Gy radiation. One hour later, cells were treated with 5 μM BC-23 and incubated for 24 h. After washed with PBS, cells were fixed in 70% cold ethanol for 15 min at -20°C, and then rehydrated in PBS for another 15 min. Cell pellets were resuspended and stained with 3 μM propidium iodide (PI, life technologies, P3566) in 0.5 mL buffer (100 mM Tris, pH = 7.4, 150 mM NaCl, 1 mM CaCl_2_, 0.5 mM MgCl_2_, 0.1% Nonidet P-40) with 1 μg/mL RNase A. The cells were incubated for 40 min at room temperature. Flow cytometric analysis was performed with an excitation wavelength of 488 nm and emission wavelength of 576 nm. Cell cycle was analyzed using FlowJo (version 9.7.5) analysis software.

### Determination of ROS production

The generation of ROS was determined using the H_2_DCFDA-based assay, according to the methods published previously [32, 33]. Briefly, H1299 cells were plated in 96-well plates at 2.5 ×10^4^ cells/well and cultured for 24 h. After twice washing with PBS, cells were incubated with 20 μM H_2_DCFDA (Cayman Chemical) at 37°C for 45 min and then treated with BC-23, radiation, or a combination of BC-23 plus radiation. The fluorescence intensity (excitation/emission = 485/535 nm) was recorded for 2 h using a Synergy H1 microplate reader (BioTek).

### Statistical Analysis

A statistical analysis was conducted using the One-way ANOVA followed by Tukey's Multiple Comparison Test (Prism 5.0, GraphPad Software). *P* values <0.05 were considered statistically significant. Average values were expressed as means ± S.D. The combined anticancer effects of BC-23 and X-ray radiation were determined using Chou and Talalay method: CI (combination index) = [(D)_1_/(Dx)_1_] + [(D)_2_/(Dx)_2_] + (D)_1_(D)_2_/(Dx)_1_(Dx)_2_, where (D)_1_ and (D)_2_ represent the doses of treatment 1 (BC-23) and 2 (radiation) that produce the x effect when used in combination, (Dx)_1_ and (Dx)_2_ are the doses of treatments 1 and 2 that produce the same x effect when used alone.

## Results

### BC-23 inhibits the binding between β-catenin and Tcf4 and the Wnt/β-catenin signaling

**Fig 1:**
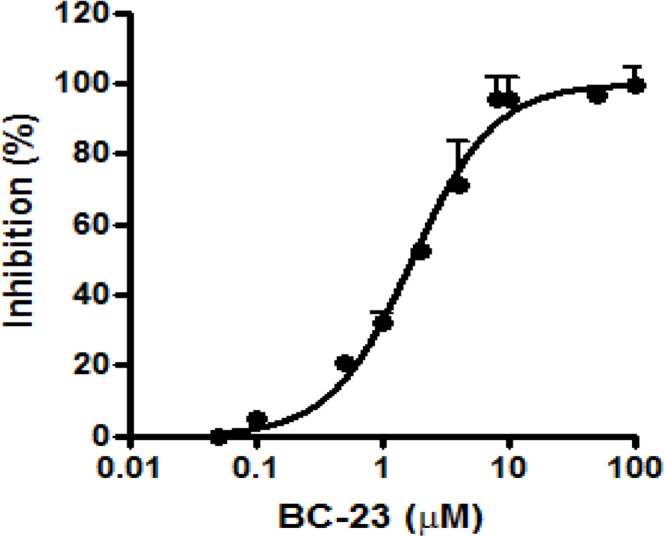
BC-23 inhibits the binding between β-catenin and Tcf4. A mixture of 500 nM β-catenin and 20 nM FITC-Tcf4_(8–30)_ was incubated in the absence or presence of the indicated concentrations of BC-23. Fluorescence polarization (FP) values were recorded after 3 h. The relative binding was calculated as (mP_T_-mP_f_) /(mP_C_-mP_f_)×100. The points represent mean ± S.D. of 3 independent experiments.

**Fig 2:**
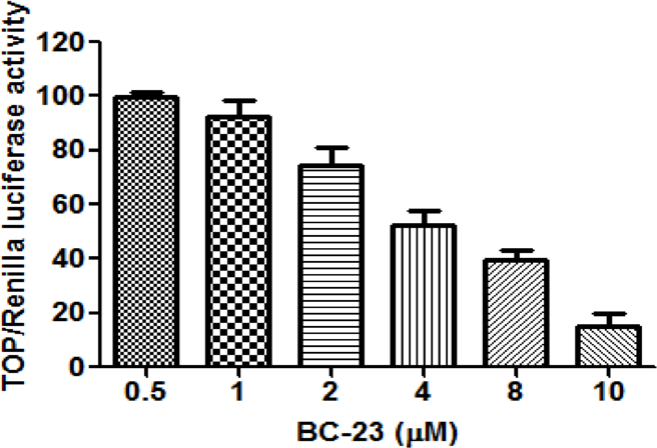
BC-23 inhibits Wnt/β-catenin signaling. H1299 cells were co-transfected with the reporter TOP-flash and the control Renilla plasmids. Four hours after transfection, cells were treated with various concentrations of BC-23 for 24 h. The TOP luciferase activities were determined and normalized to Renilla luciferase activities. BC-23 exhibited dose-dependent inhibitory activity on TOP luciferase in H1299 cells, with an IC_50_ value of 2.3 μM. The columns represent mean ± S.D. of 3 independent experiments.

We determined the direct inhibitory effects of selected compounds on the interaction between β-catenin and Tcf4 using our recently-developed cell-free competitive binding assay based on fluorescence polarization (FP). Among the compounds tested, BC-23 exhibited strong activity (IC_50_ value of 1.7 μM) against the binding between β-catenin and an FITC-labeled Tcf4_(8–30)_ probe (Fig 1). BC-21, which was reported by our group as an inhibitor of the Wnt/β-catenin pathway, was used as a positive control in FP assays. BC-21 treatment inhibited the β-catenin/Tcf-4 interactions with an IC_50_ value of 5μM [31]. The luciferase-based TOP (harboring the TCF/LEF binding site) reporter assay (used to test the inhibitory effect of BC-23 on the transcriptional activity of Wnt/β-catenin pathway in β-catenin overexpressing cancer cells) revealed that BC-23 dose-dependently inhibited TOP-luciferase activity in H1299 cells, with an IC_50_ value of 2.3 μM (Fig 2). This further suggests that BC-23 targeted the interaction between β-catenin and Tcf4 and inhibited their mediated transcriptional activity. We used the pRL Renilla-luciferase reporter as an internal control for the assay and ICRT14 (an inhibitor of β-catenin-responsive transcription) as a positive control. ICRT14 at 1 μM inhibited 38% of TOP-luciferase activity.

### BC-23 blocks the structural interaction of β-catenin with Tcf4

**Fig 3:**
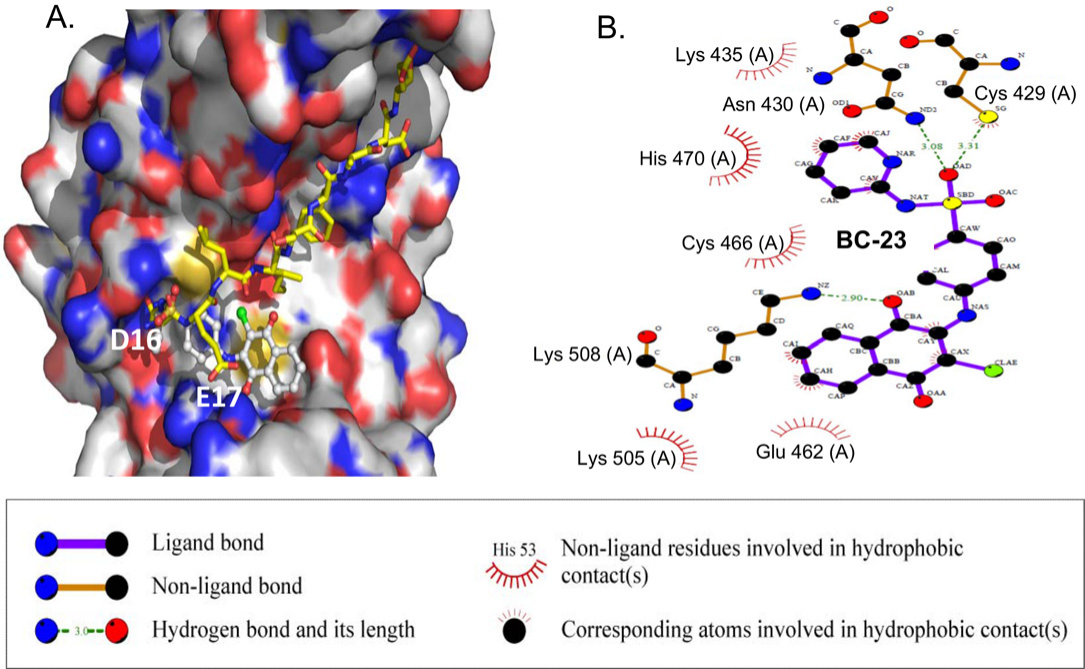
Predicted binding model for BC-23 and β-catenin. The binding models were generated by Autodock and PyMol.

We analyzed the binding conformation of BC-23 and β-catenin using molecular docking (Fig 3A). The interaction of Tcf4 Asp16 with β-catenin Lys435 is critical for their binding. Our crystal structure analysis indicated that Asp16 of Tcf4 formed a hydrogen bond with β-catenin Lys435, while Glu17 of Tcf4, which was adjacent to Asp16, formed a salt bridge with Lys508. As shown in Fig 3B, BC-23 occupied a position at Asp16 and Glu17, forming a hydrogen bond with the Asn430 side chain with distance of 3.08 Å. This presumably blocks the interaction between Tcf4 and β-catenin via Asp16 of Tcf4. In addition, β-catenin Lys508 formed another hydrogen bond with the oxygen atom of the naphthoquinone group of BC-23 with a distance of 2.90 Å. The naphthoquinone group of BC-23 also underwent hydrophobic interactions with the aliphatic parts of β-catenin Pro505. This would block the interaction of β-catenin with Tcf4 Glu17.

### BC-23 significantly enhances radiation-induced clonogenic cell death in H1299 NSCLC cells

**Fig 4:**
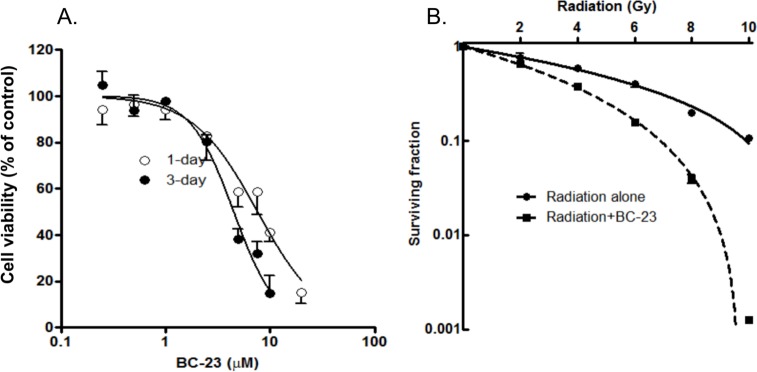
The combination of BC-23 and radiation enhances clonogenic cell death in H1299 cells. A. H1299 cells were treated with the indicated concentrations of BC-23 alone for 1 (○) and 3 (●) days. Cell viability was determined by the CellTiter Blue assay. B. Sequential exposure of H1299 cells to 2–10 Gy radiation, followed 1 h later by treatment with 3 μM BC-23 (■), dramatically increased the induction of clonogenic cell death when compared to radiation treatment alone (●). Each point represents the mean values of three separate experiments.

H1299 cells were treated with various concentrations of BC-23 for 24–72 h. At the end of each treatment, cell viability was determined with the CellTiter Blue assay. BC-23 alone exhibited moderate cytotoxicity toward H1299 cells, with IC_50_ values of 7.6 and 4.5 μM for 1 day and 3 day treatments, respectively (Fig 4A). However, sequential treatment of H1299 cells with 2–10 Gy radiation and 3μM BC-23 resulted in a 2-log increase in the clonogenic death of H1299 cells when compared with 10 Gy radiation alone (Fig 4B). The index of combination for clonogenic cell death was less than 1. This significant enhancement of clonogenic cell death in H1299 cells by the combination of BC-23 and radiation treatments indicates a radiation-enhancing effect of BC-23.

### Combining BC-23 with radiation enhances ROS generation in H1299 cells

**Fig 5:**
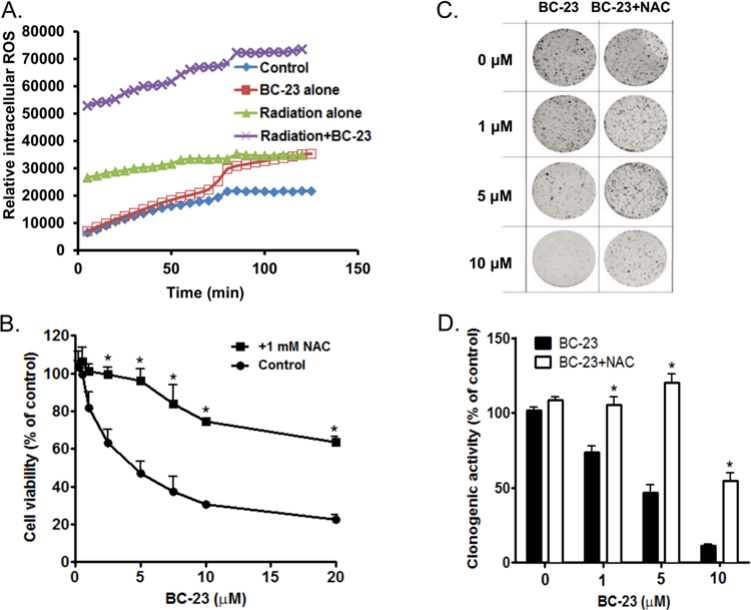
ROS generation is responsible for BC-23 induced radiation enhancement and clonogenic cell death. A. Combining 3 μM BC-23 with 10 Gy radiation (x) dramatically increased ROS generation, when compared to individual treatments alone: vehicle control (●), 3 μM BC-23 (■), and 10 Gy radiation (▲). Each value is the mean ± SD of three separate experiments. **B**-**D**, ROS-dependent cytotoxic effects of BC-23. NAC attenuates the cell death mediated by exposure of H1299 cells to the indicated concentrations of BC-23 in cytotoxicity (B) and colongenic assays (C, representative dishes of clonogenic assays and D, analyses of percentage of surviving cells). *, p<0.05 versus control.

The degree of overall oxidative stress was determined by monitoring ROS using H_2_DCFDA. We examined the intracellular levels of ROS generated over time. The ROS levels at 2 h post treatment were 2-fold higher in cells treated with the combined administration of 10 Gy radiation and 3 μM BC-23 than in cells given either treatment alone (Fig 5A). The potential role of ROS in BC-23-mediated cell killing and inhibition of colony formation was further tested using NAC (an antioxidant) to scavenge the ROS in the cells. NAC significantly abolished the cytotoxic and growth-inhibitory effects of BC-23 in H1299 cells (Fig 5B–5D).

### BC-23 induces S phase arrest in H1299 cells

**Fig 6:**
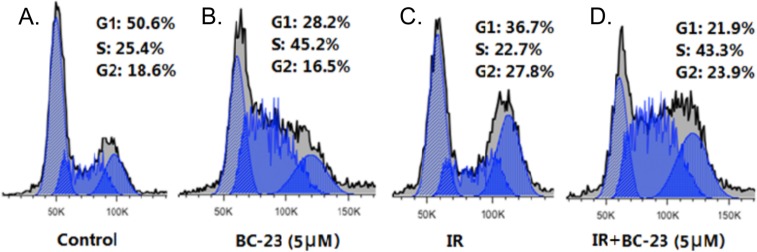
BC-23 combined with radiation enhances cell cycle arrest in the S phase and relieves radiation-induced G2/M arrest. H1299 cells were treated with vehicle control (A), 5 μM BC-23 (B), 4 Gy radiation (C), and 4 Gy radiation plus 5 μM BC-23 (D). The percentage of cells in each phase of the cell cycle was determined by propidium iodide (PI) staining and FACS analysis. The results are representative of three independent experiments.

Cell cycle arrest at the S and G2/M phases is an important mechanism that causes the cell death seen in response to treatment with a combination of radiation and anticancer drugs, including wnt/β-catenin pathway inhibitors. A 24 h treatment with 5 μM BC-23 alone significantly increased the percentage of cells (45%) in the S phase, when compared to control cells (25%). Treatment with a combination of 4 Gy radiation and 5μM BC-23 resulted in a similar percentage of H1299 cells in the S phase, but the cells were also blocked at the G2/M phase by the radiation treatment (Fig 6).

### BC-23 down-regulates the expression of c-Myc and cyclin D1 and β-catenin knockdown by CTNNB1 siRNA significantly blocked the radiosensitizing effect of BC-23

**Fig 7:**
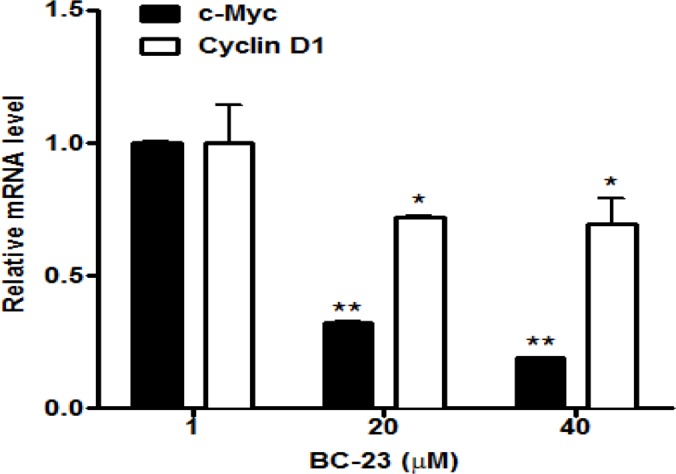
BC-23 down regulates the mRNA expression of Wnt/β-catenin target genes. The H1299 cells were treated with the indicated concentrations of BC-23. Total RNA and cDNA were prepared after a 16 h treatment. The cDNA was wuantified by real-time PCR and normalized against a vehicle control. BC-23 treatment significantly inhibited the expression of both Wnt/β-catenin target genes c-Myc and cyclin D1 at the mRNA level. The results are representative of three independent experiments. *, p<0.05 and **, p<0.01 versus control.

**Fig 8:**
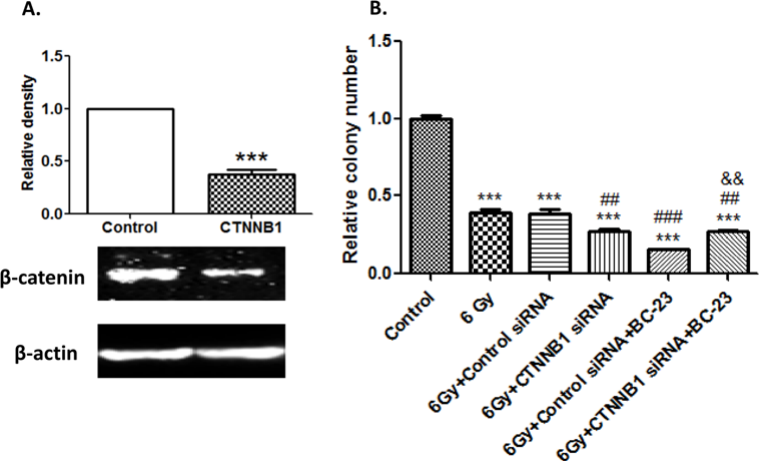
Knockdown of β-catenin by CTNNB1 siRNA significantly blocked the radiosensitizing effect of BC-23. The H1299 cells were treated with CTNNB1 or with control siRNA. Protein was prepared from a portion of the cells for western blotting and the remaining cells were used for radiosensitivity assays with 6 Gy radiation with/without BC-23. A, CTNNB1 siRNA significantly reduced the β-catenin expression. B, CTNNB1 siRNA significantly blocked the radiosensitizing effect of BC-23. The results are representative of three independent experiments. ****, p<0.001 versus control; ###, p<0.001 versus 6 Gy + control siRNA; &&, p<0.01 versus 6 Gy + control siRNA + BC-23.

We conducted further tests on the effects of BC-23 on Wnt/β-catenin/Tcf4 signaling pathway by determining its effects on the expression of c-Myc and cyclin D1, which are two important specific target genes of β-catenin/Tcf4 signaling. After a 16 h treatment with 20 and 40 μM BC-23, the mRNA expression levels of c-Myc and cyclin D1 were significantly reduced in H1229 cells (Fig 7). We examined the expression of other genes (e.g., β-actin) that were not related to regulation by β-catenin/Tcf4 interaction and their expression was not affected by BC-23. The threshold cycle values in Fig 7 were normalized to the β-actin internal reference. These data provide further evidence that BC-23 down-regulates both cyclin D1 and c-Myc, which are overexpressed in many cancer cells, including H1229 cells. We also used CTNNB1 siRNA to reduce the β-catenin expression in H1299 cells in order to determine whether the radiosensitizing effect of BC-23 depends on the β-catenin pathway. Fig 8A shows that CTNNB1 siRNA significantly reduced the β-catenin expression compared with control siRNA. Silencing of the components of the Wnt/β-catenin pathway is known to increase the radiosensitivity of cancer cells. As expected, the knockdown of β-catenin by CTNNB1 siRNA significantly enhanced the radiosensitivity of H1299 cells, whereas transfection with control siRNA did not change the radiosensitivity when compared to 6 Gy radiation alone, as shown in Fig 8B. BC-23 increased the radiosensitivity of H1299 cells treated with control siRNA. However, down regulation of β-catenin expression with CTNNB1 siRNA blocked the radiosensitizing effect of BC-23 when compared to the control siRNA group, suggesting that the radiosensitizing effect of BC-23 depends, at least in part, on the Wnt/β-catenin pathway.

## Discussion

We determined the direct inhibitory effects of selected compounds from NCI on the interaction between β-catenin and Tcf4 using our recently developed cell-free competitive binding assay based on FP [31]. Among the compounds tested, BC-23 exhibited strong activity (an IC_50_ value of 1.7 μM) against the interaction between β-catenin and an FITC-labeled Tcf4_(8–30)_ probe (Fig 1). We also used a cell-based TOP (harboring the Tef/Lef binding site) luciferase assay to test the effect of BC-23 on transcriptional activity of the Wnt/β-catenin pathway. BC-23 dose-dependently inhibited TOP activation in H1299 cells, with an IC_50_ value of 2.3 μM (Fig 2). These data suggest that BC-23 targets the β-catenin/Tcf4 interaction. Analysis of the binding conformation of BC-23 by molecular docking revealed that BC-23 occupied a position at Asp16 and Glu17 of Tcf4, forming two hydrogen bonds with Asn430 and Lys508 and hydrophobic interactions with Pro505 of β-catenin (Fig 3B). This indicated that BC-23 blocks the interaction between Tcf4 and β-catenin at Asp16 and Glu17 of Tcf4 (Fig 3A & 3B).

We tested the potential of BC-23 to act as a radiation enhancer for NSCLC H1299 cells. High levels of nuclear β-catenin have been found in many human lung cancer cells [16]. The presence of lung cancer stem cells (LCSCs) that are capable of self-renewal, proliferation, and metastasis may confer tumor resistance to radiation [4–6]. We hypothesized that administration of BC-23 might improve the action of radiotherapy on lung cancer cells through the inhibition of β-catenin/Tcf4 interaction and signaling. We first determined the cytotoxicity of BC-23 alone in H1299 cells by the CellTiter-Blue Assay. BC-23 alone exhibited moderate cytotoxicity toward H1299 cells, with IC_50_ values of 7.6 and 4.5 μM for 1 day and 3 day treatments, respectively, as shown in Fig 4A. Treatment of H1975 cells (a p53 wild type NSCLC) with BC-23 for 1 and 3 days resulted in IC_50_ values for BC-23 of 7.9 and 5.2 μM, respectively, which were values comparable to those obtained for H1299 cells. This suggests that the cytotoxic effect of BC-23 is independent of p53. Sequential treatment of the cells with 2 to 10 Gy radiation, followed 1 h later by administration of 3μM BC-23, significantly enhanced the radiation-mediated clonogenic cell killing effect in H1299 cells (Fig 4B). BC-23, in combination with 10 Gy radiation, caused a 2-log increase in the clonogenic death of H1299 cells when compared to 10 Gy radiation alone.

The potential mechanisms by which BC-23 enhances the death of NSCLC cells exposed to radiation were further explored by analyzing the cell cycle and ROS production in H1299 cells treated with BC-23 and radiation, alone or in combination. Previous study suggests that the Wnt/β-catenin signaling pathway plays a role in the regulation of cell cycle and nucleus-localized β-catenin–Tcf complex increases during S-G_2_ [34, 35]. The β-catenin activation is associated with the survival of cells with damaged DNA (caused by ROS) and with DNA repair errors [36]. BC-23, alone or combined with radiation, arrested H1299 cells in the S phase (Fig 6), when compared to the vehicle control and radiation alone. The combined treatment of BC23 and radiation produced S phase arrest and a simultaneous radiation-induced G2/M cell cycle block. These findings suggest that a delay occurs in the cell cycle transition or/and in DNA double strand break processing in the H1299 cells after co-treatment with BC-23 and radiation. The delay in DNA synthesis caused by BC-23 may worsen the survival of cells undergoing G_2_/M arrest (Fig 6) caused by exposure to radiation and the resulting ROS (Fig 5). BC-23 significantly induced ROS generation in H1299 cells in a dose-dependent manner (Fig 5A). The potential role of ROS in BC-23-mediated cell killing and clonogenic cell death was further confirmed using the antioxidant NAC to scavenge the ROS in the cells. NAC treatment significantly abolished the cytotoxic effect of BC-23 in both cytotoxicity and colony assays (Fig 5B–5D). The increased ROS induced by BC-23 may further inhibit the β-catenin/Tcf4 pathway because ROS negatively modulate the Wnt/β-catenin signal pathway through down regulation of β-catenin [37].

We obtained further confirmation of the specific effect of BC-23 on Wnt/β-catenin/Tcf4 signaling by examining the mRNA expression of c-Myc and cyclin D1, two critical downstream genes of the Wnt/β-catenin pathway. Significant inhibition of the expression of both c-Myc and cyclin D1 in H1299 cells was observed following a 16 h treatment with 20 and 40 μM BC-23 (Fig 7). Further evidence was provided by the observation that CTNNB1 siRNA reduced the β-catenin expression and significantly blocked the radiosensitizing effect of BC-23 in H1299 cells (Fig 8). Taken together, the ligand- and pathway-specific cell-free and cell-based assays, as well as *in silico* molecular docking results, demonstrated that BC-23 is a new inhibitor of β-catenin/Tcf4 that specifically targets the β-catenin/Tcf4 interaction and inhibits their transcriptional activity [38].

## Conclusion

We have identified and characterized a new small molecule BC-23 that targets the interaction of β-catenin/Tcf4 and inhibits their transcriptional activity. BC-23 down regulates two important Wnt/β-catenin pathway genes: c-Myc and cyclin D1. Treatment of H1299 NSCLC cells with a minimally cytotoxic concentration of BC-23 dramatically enhanced the clonogenic cell death in response to radiation. S-phase arrest, ROS generation, and relieving G2/M arrest are parts of the mechanism underlying BC-23-mediated radiation enhancement. BC-23 represents a promising new inhibitor of the Wnt/β-catenin pathway and a radiation enhancer.
